# Assessing Foveal Structure in Individuals with *TYR* R402Q and S192Y Hypomorphic Alleles

**DOI:** 10.1016/j.xops.2021.100077

**Published:** 2021-11-17

**Authors:** Gelique D. Ayala, Rachel E. Linderman, Robert K. Valenzuela, Erica N. Woertz, Murray Brilliant, Sergey Tarima, Joseph Carroll

**Affiliations:** 1 School of Medicine, Medical College of Wisconsin, Milwaukee, Wisconsin; 2 Ophthalmology & Visual Sciences, Medical College of Wisconsin, Milwaukee, Wisconsin; 3 Center for Precision Medicine, Marshfield Clinic Research Institute, Marshfield, Wisconsin; 4 Waisman Center, University of Wisconsin-Madison, Madison, Wisconsin; 5 Division of Biostatistics, Institute of Health and Equity, Medical College of Wisconsin, Milwaukee, Wisconsin; 6 Cell Biology, Neurobiology & Anatomy, Medical College of Wisconsin, Milwaukee, Wisconsin

**Keywords:** Foveal avascular zone, Foveal development, Foveal morphology, Genetics, Pigmentation, Retina, Retinal imaging, FAZ, foveal avascular zone, SD, standard deviation

## Abstract

**Purpose:**

To assess the impact of two *TYR* hypomorphic alleles (R402Q and S192Y) on foveal pit and foveal avascular zone (FAZ) morphology.

**Design:**

Prospective, cross-sectional study.

**Participants:**

A total of 164 participants with normal vision (67 male and 97 female; mean ± standard deviation [SD] age = 30.5 ± 12.8 years) were recruited.

**Methods:**

Sequencing of more than 100 pigmentation-related genes was performed, and results were reviewed for the presence or absence of the *TYR* polymorphisms R402Q (rs1126809) and S192Y (rs1042602). Volumetric scans of the macula were obtained for each participant using OCT, and retinal thickness maps were analyzed using custom software. OCT angiography was used to image the FAZ, which was manually segmented and measured. Linear mixed model analysis was used to assess associations between genotype and foveal pit morphology.

**Main Outcome Measures:**

Foveal pit depth, diameter, volume, and FAZ area in relation to the presence of hypomorphic alleles R402Q and S192Y on the *TYR* gene.

**Results:**

Heterozygosity for the *TYR* R402Q allele was associated with decreased pit diameter (*P =* 0.0094) and decreased FAZ area (*P =* 0.025). Homozygosity for the *TYR* R402Q allele was associated with reduced pit volume (*P =* 0.0005), decreased pit depth (*P =* 0.007), reduced pit diameter (*P =* 0.0052), and reduced FAZ area (*P =* 0.0012). Homozygosity for *TYR* S192Y was associated with reduced FAZ area (*P =* 0.016). Heterozygosity for the *TYR* S192Y allele was not associated with differences in foveal pit depth, diameter, volume, or FAZ area (*P* > 0.05).

**Conclusions:**

Although the role of the *TYR* R402Q and S192Y hypomorphic alleles in albinism remains controversial, our data suggest that these variants contribute to the extensive inter-individual variability in foveal morphology in the normal population. Our results contribute to the evolving picture of the relationship between ocular pigmentation and foveal morphology.

The fovea is a highly specialized region of the primate retina and is responsible for high acuity vision. The foveal region is characterized by a foveal avascular zone (FAZ), increased cone photoreceptor density, and the characteristic pit (“fovea” is Latin for “pit”). Although the steps involved in development of the fovea have been well elucidated,^1^^,^^2^ one aspect of foveal anatomy that is not well understood is the enormous variation in the size of the pit and FAZ. For example, Wilk et al^3^ showed that pit volume varied by 11-fold among 64 individuals with normal vision.^3^ Chui et al^4^ also found significant inter-individual variability, with a 2.5-fold variation in pit depth among 11 individuals with normal vision. Additionally, in a study of 110 normal foveae, Tick et al^5^ reported large coefficient of variations for both pit depth (16.7%) and foveal inner retinal area (22.1%). Finally, Linderman et al^6^ found FAZ area varied by 9-fold in a study with 350 normal foveae. Although conditions such as premature birth have been associated with smaller-sized pits and FAZs,^7^, ^8^, ^9^ the factors underlying normal variation in pit size remain incompletely understood.

Insight on this issue comes from data on individuals with albinism. These individuals have absent or reduced retinal melanin and lack a fully developed foveal pit (i.e., foveal hypoplasia).^10^^,^^11^ A further link between pigmentation and foveal anatomy has been suggested by literature comparing normal foveal anatomy between different racial groups. It has been shown that Black individuals have significantly deeper and broader pits compared with their White counterparts.^12^^,^^13^ Although there is no evidence for racial differences in retinal pigment epithelium melanin, there are racial differences in choroidal melanin.^14^ If racial differences in choroidal melanin result in differences in foveal morphology, it stands to reason that individual variation in choroidal melanin could contribute to the described variation in foveal morphology.

One of the key enzymes in the melanin biosynthesis pathway is tyrosinase, encoded by the *TYR* gene. Although *TYR* polymorphisms are known to cause autosomal recessive oculocutaneous albinism, there are some common hypomorphic alleles whose involvement in albinism is controversial.^15^, ^16^, ^17^, ^18^ Despite the controversy surrounding their pathogenicity, it has been shown that these alleles are associated with reduced enzymatic activity of tyrosinase.^19^ For example, the R402Q variant (rs1126809) has been reported to have 25% to 67% enzymatic activity relative to wild-type at normal body temperature,^19^^,^^20^ whereas the S192Y variant (rs1042602) results in 60% enzymatic activity.^21^ The allele frequency of R402Q is 25% worldwide, but higher in European (non-Finnish) populations (36.4%).^22^ Likewise, the allele frequency of S192Y is 17.65% worldwide and 27.28% in European (non-Finnish) populations.^22^ The high frequency of these alleles, together with their mechanism of action, make them intriguing candidates for contributors to the normal variability in the size of the human fovea. We sought to examine the relationship between *TYR* genotype and the size of the pit and FAZ in individuals with normal vision.

## Methods

### Participants

This study was approved by the Institutional Review Boards at the Medical College of Wisconsin (PRO23898) and followed the tenets of the Declaration of Helsinki. All participants provided informed consent following discussion of the risks of participation. A legal guardian provided consent for participants younger than 18 years of age. All participants completed an ocular health questionnaire and underwent color vision testing using the Neitz test of Color Vision (Western Psychological Services).^23^ By using the National Institutes of Health’s racial and ethnic categories, each participant was given the option to report their race and ethnicity. Exclusion criteria for the study included being aged less than 5 years, color vision deficits, and self-reported ocular, systemic disease, or being born prematurely.

### Genetic Testing

Samples were sent to the Marshfield Clinic Research Foundation. Genomic DNA was extracted from the submitted specimen followed by enzymatic fragmentation and enrichment using SureSelect Custom DNA Target Enrichment Probes ^QXT^ (Agilent Technologies) that targeted more than 100 pigmentation-related genes. Size selection and cleanups were performed using KAPA Pure Beads (Roche); size selection and libraries were visualized on an Agilent 2100 Bioanalyzer. Libraries were quantified using KAPA Library Quantification Kit (Illumina) and visualized on a LightCycler 480 (Roche). Samples were 10-plexed/cartridge and pair-end sequenced (75 base pairs) on Miseq (Illumina). The sequenced data were aligned (GRCh37), variant-called, and annotated using SureCall v4.0, v4.1, v4.2 (Agilent Technologies).

### Foveal Morphology Imaging and Analysis

Axial length was measured using the IOL Master 500 (Carl Zeiss Meditec). OCT imaging was performed on the right eye for each participant, using the Cirrus 6000 high-definition OCT (Carl Zeiss Meditec). Each volume scan was a nominal 6 × 6-mm scan consisting of 128 B-scans (512 A-scans/B-scan).

Foveal pit morphology was analyzed using a previously published difference of Gaussian algorithm.^3^ The retinal pigment epithelium internal limiting membrane thickness data file for each volume was imported into the software and was bilinearly interpolated to 512 × 512 pixels. The volume was then segmented into 1° radial slices passing through the foveal center (determined using the FoveaFinder algorithm on the Cirrus software). The thickness profile of each slice was fit to a difference of Gaussian model fit where all parameters varied freely to account for potential asymmetry of the retinal height and slope on either side of the fovea. Foveal metrics were measured on the basis of the Gaussian model fit values for each radial slice. Foveal pit depth was defined as the difference of the average maximum height and at the retinal thickness minimum. Foveal pit diameter was defined as the distance between the 2 maximum heights. Foveal pit depth and diameter were then averaged across all radial slices.

To calculate the foveal volume, the different fits were all plotted together, and a fifth order polynomial was calculated to create a 1-dimensional contour filter that was then applied at the fovea (defined as the area between the maximum height). Once the smoothing was complete, the surface, or the top of the fovea, was calculated by drawing a line between the peaks of the 2 Gaussian curve fits. Once this was done, the surface was then overlaid onto the thickness measurements and the macular thickness measurements were subtracted from the surface to estimate the volume filling that space.

### Foveal Avascular Zone Imaging and Analysis

With the Optovue’s Avanti system, OCT angiography was done on all but 6 participants. For all but 2 subjects who were imaged, 2 to 5 angiograms centered on the fovea were taken at a nominal scan size of 3 × 3 mm. For the 2 subjects, a single angiogram was used. For each angiogram, 2 volumes consisting of 304 B-scans at 304 A-scans/B-scan were acquired; 1 volume had a horizontal fast scanning axis, and the other volume had a vertical fast scanning axis. The 2 volumes were registered (AngioVue software version: 2018.1.0.43) to create a single angiogram volume with reduced motion artifacts and better signal-to-noise ratio. For each volume, the full-thickness slab (inner limiting membrane to 9 μm above the outer plexiform layer) was extracted. The 2 to 5 extracted images were then averaged together using bUnwarpJ^24^ in FIJI^25^ to further increase the signal-to-noise ratio and cropped to 304 × 304 pixels. The averaged image was used for further FAZ analysis.

Using ImageJ’s multipoint tool,^26^ a single observer (R.E.L.) segmented each FAZ. The segmentation coordinates were entered into a custom MATLAB script that has been previously described.^27^^,^^28^ The area of the FAZ (or the largest avascular area if the FAZ was fragmented^29^) was computed in pixels using the *polyarea* function in MATLAB. This was converted to mm^2^ by multiplying the nominal image scale (9.87 μm/pixel) by the ratio of the participant’s measured axial length to the axial length assumed by the Avanti system (23.95 mm).^30^

### Statistical Analysis

The objective was to investigate how foveal metrics differ between individuals with and without R402Q or S192Y, assuming other individual characteristics related to the metrics are similar. Furthermore, gene effects were defined as the differences in foveal metrics among participants with or without the R402Q or S192Y polymorphism. For each metric, Pearson median skewness was calculated to assess the distribution of the data. If the skewness was ≥ 0.4, a natural logarithmic transform was performed on the data and used for the following models. If the median skew was < 0.4, the raw data were used. To analyze the effect of R402Q and S192Y in our population, we used a linear mixed model to control for different demographic factors that have been shown to impact foveal morphology (R, version 4.0.3; R Foundation for Statistical Computing^31^). The following factors were used as predictors: number of R402Q alleles, number of S192Y alleles, race, age, sex, and axial length. The S192Y and R402Q status were each listed as not having the polymorphism (0 polymorphism), heterozygous (1 polymorphism), or homozygous (2 polymorphisms). The following factors were outcomes: foveal pit depth, diameter, volume, and FAZ area. Once the initial model was created, nonsignificant variables were dropped (*P* > 0.05), and the model was rerun with only the significant variables in the model to acquire the final fit.

## Results

### Participant Characteristics

**Figure 1:**
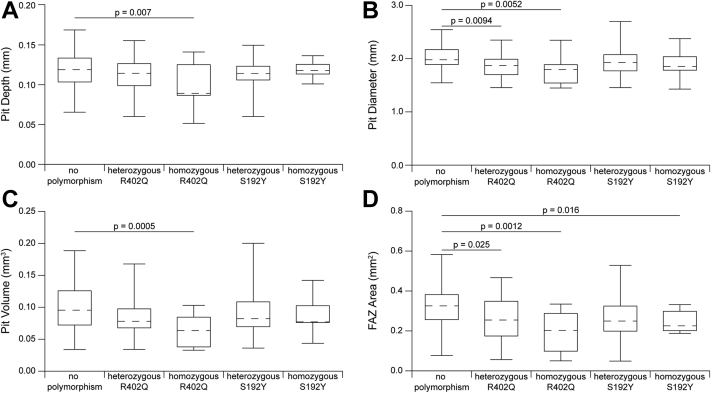
Comparisons of foveal and foveal avascular zone (FAZ) morphology metrics based on the *TYR* hypomorphic allele status. The average for each group is represented by the dashed line, the boxes represent the first to third quartiles, and the whiskers represent the minimum and maximum values for each group. Based on our mixed linear model, (**A**) pit depth, (**B**) pit diameter, (**C**) pit volume, and (**D**) FAZ area were significantly reduced in participants homozygous for R402Q. Participants heterozygous for R402Q had significantly smaller pit diameters and FAZ areas, where participants homozygous for S192Y had significantly smaller FAZ areas. Participants heterozygous for both R402Q and S192Y are included in both heterozygous categories for all metrics.

There were 164 participants (67 male and 97 female), with a mean (± standard deviation [SD]) age of 30.5 ± 12.8 years. Self-reported ethnicity included 112 White participants, 3 American Indian/Alaskan native, 24 Asian, 1 native Hawaiian/Pacific Islander, 14 Black, 8 mixed-race participants, and 2 participants who did not report race. Across our participants, the average (± SD) foveal pit depth was 0.116 ± 0.021 mm, the average (± SD) foveal pit diameter was 1.936 ± 0.249 mm, the average (± SD) pit volume was 0.091 ± 0.036 mm^3^, and the average (± SD) FAZ area was 0.276 ± 0.111 mm^2^. Summarized quantitative data are shown in Figure 1.

### *TYR* Genotype

The average read depth for the analyzable target regions of *TYR* was 187.24 ± 84.97 (range, 40–578). Nine participants were homozygous for *TYR* R402Q, and 12 participants were homozygous for *TYR* S192Y. Forty-one participants were heterozygous for only R402Q, 25 participants were heterozygous for only S192Y, and 19 participants were heterozygous for both R402Q and S192Y. There were 58 participants with no copies of the R402Q or S192Y alleles. All 9 participants homozygous for R402Q (4 male, 5 female) and all 12 participants homozygous for S192Y (5 male, 7 female) identified themselves as White. The overall allele frequency for R402Q and S192Y was 23.8% and 20.7%, respectively. As both polymorphisms are known to have a higher allele frequency in people from Northern Europe, we further examined the frequency based on self-reported race. White participants in our study population had an allele frequency of 31.7% and 27.7% for R402Q and S192Y, respectively. Black individuals had an allele frequency of 3.5% (R402Q) and 7.1% (S192Y), whereas individuals from other racial backgrounds had an allele frequency of 8.1% (R402Q) and 4.1% (S192Y). These racial differences in allele frequency are consistent with previous studies.^22^^,^^32^

### Effect of R402Q and S192Y on the Foveal Region

Genetic status and foveal morphology are summarized in Table 1. As both foveal volume and diameter had skewed distributions (Pearson median skew: 0.8 and 0.4, respectively), these data were transformed using the natural log, and the linear mixed model was run on the transformed data. Linear mixed model analysis used the following fixed variables: race, ethnicity, R402Q genotype, S192Y genotype, and sex. The continuous variables used were age and axial length.

**Table 1:** Summary of Foveal Morphology Metrics and FAZ Area Based on Genetic Status (Mean ± SD)

*TYR* Status	Depth (mm)	Diameter (mm)	Volume (mm^3^)	FAZ Area (mm^2^)
No mutation	0.119 ± 0.023 n = 58	2.015 ± 0.226 n = 58	0.100 ± 0.038 n = 58	0.321 ± 0.108 n = 56
Heterozygous R402Q	0.115 ± 0.022 n = 41	1.857 ± 0.203 n = 41	0.083 ± 0.032 n = 41	0.264 ± 0.112 n = 39
Homozygous R402Q	0.100 ± 0.028 n = 9	1.773 ± 0.280 n = 9	0.063 ± 0.026 n = 9	0.191 ± 0.105 n = 9
Heterozygous S192Y	0.117 ± 0.012 n = 25	1.983 ± 0.298 n = 25	0.099 ± 0.040 n = 25	0.273 ± 0.113 n = 24
Homozygous S192Y	0.119 ± 0.010 n = 12	1.900 ± 0.267 n = 12	0.085 ± 0.027 n = 12	0.244 ± 0.053 n = 11
Heterozygous R402Q and S192Y	0.109 ± 0.022 n = 19	1.901 ± 0.245 n = 19	0.084 ± 0.029 n = 19	0.234 ± 0.098 n = 19
All participants	0.116 ± 0.021 n = 164	1.936 ± 0.249 n = 164	0.091 ± 0.036 n = 164	0.276 ± 0.111 n =158

FAZ = foveal avascular zone; SD = standard deviation.

**Figure 2:**
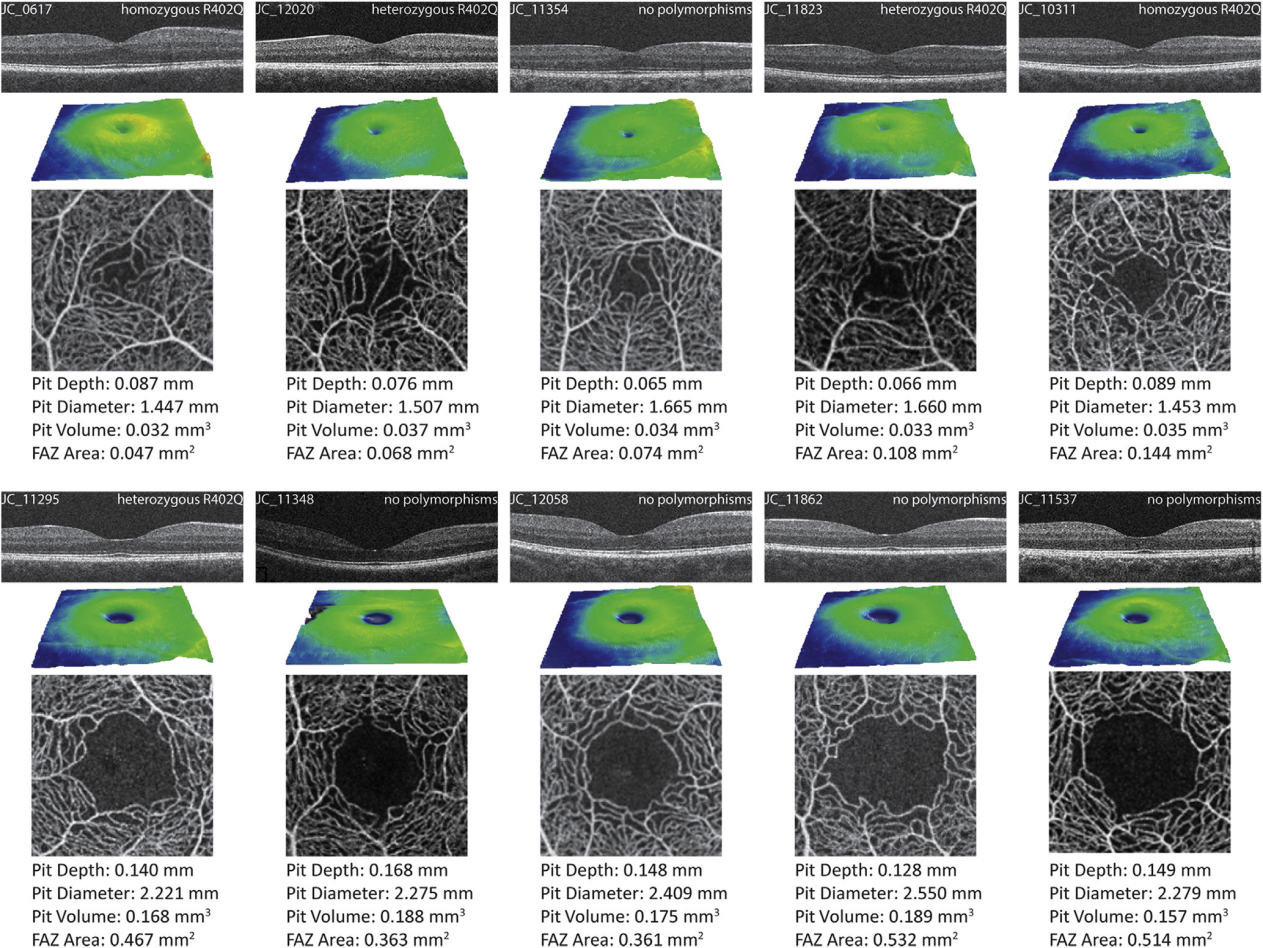
Images and measurements from eyes with the smallest and largest foveae based on a composite ranking. To create the composite ranking, each eye was rank ordered for each of the 4 metrics (pit depth, pit diameter, pit volume, and FAZ area), and their total rank was calculated by summing their ranks from each metric. The eyes with the lowest composite score were deemed to have the smallest fovea (top rows) and those with the highest composite score were deemed to have the largest fovea (bottom rows). For each participant, their OCT B-scan, macular thickness map, and averaged OCT angiography images are shown along with their measurements of pit depth, diameter, volume, and FAZ area. FAZ = foveal avascular zone.

Results showed significant reductions in all foveal and FAZ metrics for those homozygous for R402Q. There were significant reductions in diameter and FAZ area in those heterozygous for R402Q, whereas those homozygous for S192Y had significantly decreased FAZ areas. Those heterozygous for S192Y had no significant difference in foveal or FAZ morphology (Table S1, available at www.ophthalmologyscience.org). Examples of the subjects with the smallest and largest foveal metrics and FAZ areas are shown in Figure 2. Black race was associated with larger pit volumes (*P =* 0.038), diameters (*P =* 0.045), and larger FAZ areas (*P =* 0.031). White race, Asian race, and younger age were associated with smaller FAZ areas (*P =* 0.0005, *P =* 0.015, *P =* 0.0047, respectively). Longer axial length was associated with smaller pit depths (*P =* 0.0001) and smaller pit volumes (*P =* 0.0029), whereas male sex was associated with smaller FAZ areas (*P =* 0.0004). Predictors and associations for our complete model are summarized in Table S1 (available at www.ophthalmologyscience.org).

Using only the significantly associated variables (Table 2) for pit depth, linear mixed model analysis showed that individuals homozygous for *TYR* R402Q had pits that were 0.020 mm shallower than those without R402Q polymorphism (*P =* 0.007). Likewise, we found that the individuals homozygous for *TYR* R402Q had pits that were reduced in volume by 35% in comparison with those without the R402Q polymorphism (*P =* 0.0005). The analysis for pit diameter showed individuals heterozygous for *TYR* R402Q had diameters reduced by 5%, whereas participants homozygous for *TYR* R402Q had diameters reduced by 10% in comparison with those with no R402Q alleles (*P =* 0.0094 and *P =* 0.0052, respectively). Compared with those with no R402Q alleles, individuals heterozygous for *TYR* R402Q had FAZ areas that were 0.036 mm^2^ smaller, while those homozygous for *TYR* R402Q had FAZ areas that were 0.111 mm^2^ smaller (*P =* 0.025 and *P =* 0.0012, respectively). Finally, subjects homozygous for *TYR* S192Y also had reduced FAZ areas by 0.038 mm^2^ compared with those with no *TYR* S192Y mutations (*P =* 0.016).

**Table 2:** Effect Size of the Predictors Found to Be Significant in the Final Linear Regression Model

Predictors	Depth (mm)		Diameter (%)∗		Volume (%)∗		FAZ Area (mm^2^)	
	Effect Size	P	Effect Size	P	Effect Size	P	Effect Size	P
Heterozygous R402Q	–	NS	−4.79	0.0094	–	NS	−0.036	0.025
Homozygous R402Q	−0.020	0.007	−9.95	0.0052	−34.70	0.0005	−0.111	0.0012
Heterozygous S192Y	–	NS	–	NS	–	NS	–	NS
Homozygous S192Y	–	NS	–	NS	–	NS	−0.038	0.016
Male	–	NS	–	NS	–	NS	−0.060	0.0004
White race	–	NS	–	NS	–	NS	−0.059	0.0005
Asian race	–	NS	–	NS	–	NS	−0.020	0.015
Black race	–	NS	11.06	0.0028	34.04	0.0022	0.091	0.032
Age	–	NS	–	NS	–	NS	0.002	0.0049
Axial length (mm)	−0.0058	0.0001	–	NS	−6.93	0.003	–	NS

FAZ = foveal avascular zone; NS = not significant (in final linear mixed effects regression model); – = no significant effect.

∗ Data were transformed using the natural log; effect size is reported as a percentage.

## Discussion

This study examined the association between *TYR* R402Q and S192Y hypomorphic alleles and foveal structure. Compared with participants with no R402Q or S192Y alleles, those heterozygous for the *TYR* R402Q hypomorphic allele had significantly smaller foveal diameters (7% decrease on average) and FAZ areas (21% decrease on average). Participants homozygous for R402Q showed even larger effects, with a 16% reduction in pit depth, 12% reduction in foveal diameter, 37% reduction in foveal volume, and 41% reduction in FAZ area compared with those with no R402Q or S192Y alleles. Only the FAZ area was significantly reduced in subjects homozygous for *TYR* S192Y (by 24% on average). These effects in the raw data were generally consistent with the effects observed using the linear mixed model analysis.

Additional support for our findings comes from a recent genome-wide association study of inner retinal morphology using the UK Biobank.^33^ The authors found that the S192Y variant was associated with increased retinal thickness (i.e., a shallower foveal pit), whereas R402Q was not associated with retinal thickness in their cohort. The different metrics used in Currant et al^33^ may explain the contrasting results observed in our study. That study assessed the average retinal nerve fiber layer thickness and GCL-IPL layer thickness across the Macula6 grid and the total retinal thickness at the fovea, whereas our study assessed the shape of the foveal pit and the FAZ area (using OCT angiography). Although our results from the full model analysis show the same overall trend as our condensed analysis using the model with significant variables, the full model showed that the S192Y variant just missed statistical significance in its association with FAZ area in the heterozygous state (*P =* 0.052) and was significant in the homozygous state (*P =* 0.015) state (Tables S1 and S2, available at www.ophthalmologyscience.org). Nevertheless, both studies support a role for tyrosinase activity in the development of the size and shape of the fovea, though further work examining the contribution of the S192Y and R402Q alleles is warranted (especially related to any additive effects they may have when present in *cis* vs. *trans*).

There are important limitations to this study. First, there were a relatively small number of participants who were homozygous for R402Q (n = 9) or S192Y (n = 12). This may also explain the differences in results when we compare the results here with those of Currant et al.^33^ Furthermore, 10 children aged less than 16 years were included in this study, which could potentially confound metrics of pit morphology for their respective genetic group (since foveal development may continue after birth^34^, ^35^, ^36^). However, the statistical analyses accounted for foveal differences due to age when examining the effects of the *TYR* hypomorphic alleles. In addition, although previous research has shown that these alleles can result in decreased melanin in the retina, choroid, iris, skin, or hair,^19^, ^20^, ^21^ we did not do any qualitative or quantitative assessment of melanin in our participants. We also did not account for any potential environmental factors that may impact retinal development of these individuals. Finally, we examined only 2 hypomorphic variants on the *TYR* gene. Other hypomorphic alleles in the *TYR* and *OCA2* genes have been reported.^16^^,^^19^^,^^37^, ^38^, ^39^ Further assessment of how these alleles impact foveal morphology, as well as continued identification of other potential hypomorphic alleles in genes implicated in the pigmentation pathway, would be the next steps in understanding the large variability of normal foveal anatomy.

In conclusion, the results presented expand our understanding of the normal variability in foveal morphology. On one end of the pigmentation spectrum are Black individuals, who have deeper and broader foveal pits.^12^^,^^13^ On the other end are patients with albinism, who range from having an absent foveal pit to a shallow pit.^11^ White individuals and individuals from other racial backgrounds fall in between these extremes. There are likely other genes that regulate the formation of the foveal pit; however, our data suggest that the variations we see within racial groups may be driven (at least in part) by hypomorphic alleles in *TYR.*
