# Supplementary material for: Assessing Foveal Structure in Individuals with TYR R402Q and S192Y Hypomorphic Alleles
Source: Ophthalmol Sci. 2021 Nov 17;1(4):100077. doi: 10.1016/j.xops.2021.100077 (PMC9560529; doi:10.1016/j.xops.2021.100077)
Supplement: Table S1 [file mmc1.pdf]

Supplementary Table 1. Effect size based on full linear mixed effects linear regression model

Predictors	Depth (mm)		Diameter (%)[a]		Volume (%)[a]		FAZ Area (mm^2)	
	Effect Size	p	Effect Size	p	Effect Size	p	Effect Size	p
**Heterozygous R402Q**	-0.0068	0.24	-4.16	0.010	-13.56	0.062	-0.026	0.024
**Homozygous R402Q**	-0.022	0.0078	-7.79	0.0057	-33.02	0.0006	-0.099	0.0011
**Heterozygous S192Y**	-0.0046	0.28	1.46	0.89	2.05	0.78	-0.024	0.052
**Homozygous S192Y**	-0.42	0.82	-1.61	0.14	-8.84	0.29	-0.026	0.015
**Male**	0.0030	0.86	-3.70	0.15	-7.94	0.064	-0.063	0.0004
**White race**	0.0020	0.91	-2.37	0.034	-3.60	0.22	-0.055	0.0005
**Asian race**	0.0039	0.29	1.19	0.31	2.24	0.096	-0.017	0.015
**Black race**	0.0089	0.18	9.49	0.045	28.98	0.038	0.10	0.031
**Age**	0.00039	0.99	-0.18	0.57	-0.29	0.40	0.0020	0.0047
**Age^2 ($years^2$)**	-3.8E-06	0.59	0.0016	0.74	0.0025	0.88	-3.48E-06	0.94
**Ethnicity (Not Hispanic)**	-0.0023	0.79	-0.63	0.86	-0.94	0.98	-0.041	0.15
**Axial Length (mm)**	-0.0064	0.0001	0.32	0.70	-7.39	0.0029	0.011	0.10

a – data were transformed using the natural log, effect size is reported as a percentage
